# Increased Risk of Metabolic Syndrome in Antidepressants Users: A Mini Review

**DOI:** 10.3389/fpsyt.2018.00621

**Published:** 2018-11-28

**Authors:** Carla Gramaglia, Eleonora Gambaro, Giuseppe Bartolomei, Paolo Camera, Maira Chiarelli-Serra, Luca Lorenzini, Patrizia Zeppegno

**Affiliations:** ^1^Psychiatry Ward, University Hospital Maggiore della Carità, Novara, Italy; ^2^Department of Translational Medicine, Institute of Psychiatry, Università del Piemonte Orientale, Novara, Italy

**Keywords:** metabolic syndrome, depression, antidepressants, cardiometabolic disease, review, preferred reporting items for systematic reviews and meta-analyses (PRISMA) statement

## Abstract

Mounting evidence has shown that the risk of metabolic syndrome (MetS) is substantially overlapping in the diagnostic subgroups of psychiatric disorders. While it is widely acknowledged that patients receiving antipsychotic medications are at higher risk of MetS than antipsychotic-naive ones, the association between antidepressants and MetS is still debated. The goal of our mini review was to analyse the relationship among depressive symptoms, antidepressant use and the occurrence of MetS. Adhering to PRISMA guidelines, we searched MEDLINE, reference lists and journals, using the following search string: (((“Mental Disorders”[Mesh]) AND “Metabolic Syndrome”[Mesh]) AND “Antidepressive Agents”[Mesh]), and retrieved 36 records. Two reviewers independently assessed records and the mini review eventually included the data extracted from 8 studies. The Newcastle-Ottawa Scale was used to assess the quality of the selected studies. Overall, the results of the mini review seem to support the association among depressive symptoms, antidepressants therapy and MetS. Except for H1-R high-affinity ones, the relationship between antidepressants and MetS still needs to be clarified. Considering the widespread prescription of antidepressants, both on behalf of psychiatrists and primary care physicians, further research on this topic is recommended.

## Introduction

Metabolic syndrome (MetS) is a cluster of obesity, insulin resistance, hypertension, impaired glucose tolerance or diabetes, hyperinsulinemia, elevated triglycerides and low high-density lipoprotein (HDL) concentrations (1, 2). A syndrome can be regarded as “a clustering of factors that occur together more often than by chance alone and for which the cause is often uncertain” (3). According to the International Diabetes Federation (IDF) definition, MetS is characterized by central adiposity plus two or more of the following four factors (4): (1) raised concentration of triglycerides: ≥150 mg/dl (1.7 mmol/l) or specific treatment for this lipid abnormality; (2) reduced concentration of HDL cholesterol: 40 mg/dl (1.03 mmol/l) in men and 50 mg/dl (1.29 mmol/l) in women, or specific treatment for this lipid abnormality; (3) raised blood pressure: systolic blood pressure ≥130 mmHg or diastolic blood pressure ≥85 mmHg or treatment of previously diagnosed hypertension; and (4) raised fasting plasma glucose concentration ≥100 mg/dl (5.6 mmol/l) or previously diagnosed type 2 diabetes. Furthermore, the IDF lists ethnic group–specific thresholds for waist circumference to define central adiposity (5).

National Cholesterol Education Program Adult Treatment Panel III (NCEP ATP III) criteria require three out of five factors to establish the diagnosis of MetS, i.e., abdominal obesity (waist circumference >88 cm for women or > 102 cm for men), increased triglycerides (≥150 mg/dL), reduced HDL cholesterol (< 50 mg/dL for women or < 40 mg/dl for men), high blood pressure (≥130/85) (6) and high fasting glucose (≥100 mg/dL) (7).

A vast body of literature (8–16) has pointed to a major role of mental illness (especially bipolar disorder, depression, anxiety, and suicidal ideation) in the future development of MetS and associated diseases. A variety of factors may be responsible for MetS in patients suffering from mental illnesses, such as lifestyle, diet, tendency to insulin resistance, and medication side-effects, especially those of antipsychotics (17). Antipsychotics, mostly second generation ones (SGAs) such as clozapine, olanzapine, and risperidone, seem to be involved in the development of MetS (18, 19). Albeit the pathophysiology of SGAs-induced metabolic alterations is not yet fully elucidated, increased food intake, weight gain, hyperglycemia, lipid accumulation in adipose cells and liver are hallmarks of this problem (19).

Several studies (20–23) have shown a high comorbidity between major depressive disorder (MDD) and MetS (24, 25). Depression can cause a 2-fold increase in the risk of MetS in the general population, probably due to poor health-related behaviors (26). Furthermore, independent of the psychiatric disorder diagnosis, antidepressants may have a direct impact on MetS (27), and overall negative consequences for cardiometabolic health (28–31).

While prescription of antidepressant medication is increasing (30) and there is evidence of weight gain induced by antidepressants (31), the association between Mets and antidepressants still remains only partially understood. The introduction of tricyclic antidepressants (TCAs) in the late 1960s, followed by that of selective serotonin re-uptake inhibitors (SSRIs) in the 1980s, together with the increase of long-term prescriptions (in the 1990s and 2000s) and to the more recent use of higher doses of antidepressants, have contributed to a tendency toward over prescribing of antidepressants (32). Although there is much evidence supporting the association between MetS dysregulations and the use of TCAs, particularly abdominal obesity (33, 34), the effects of SSRIs on MetS are far less clear (35, 36).

Considering the possible role of mental illness in the future development of MetS and the need to clarify the impact of antidepressant treatment on MetS, the aim of this mini-review was to address the relationship among depressive symptoms, antidepressant use and the occurrence of MetS.

## Methods

Adhering to PRISMA guidelines, a literature search was conducted in MEDLINE on 13 February 2018, using the following search string: (((“Mental Disorders”[Mesh]) AND “Metabolic Syndrome”[Mesh]) AND “Antidepressive Agents”[Mesh]). The search was restricted to the English language. To be included in the mini review, papers had to be cross-sectional or cohort studies designed with the purpose of analyzing the association between MetS, depressive symptoms or antidepressants therapy. Two reviewers (CG and EG) independently triaged the titles and then the abstracts to exclude those that were clearly inappropriate. Possible disagreement between reviewers was resolved by joint discussion with a third review author (PZ). Reasons for the exclusion of papers from the review were reported in the PRISMA flow diagram.

After selection of the relevant studies, reviewers extracted and tabulated data using a standard form (Table 1). Extracted data included country of origin, research objectives, databases and period assessed, study design, participants' features. Some data were tabulated, while other, including those about educational and occupational level, socio-demographic data collection methods, lifestyle data collection methods, anthropometric measurements (including waist circumference and Body Mass Index [BMI]), were reported in the text description in the Results section.

**Table 1:** Narrative synthesis of the studies included in the mini-review.

**Authors**	**Country and study period**	**Design and number of participants**	**Participants' features (mean age, gender, % M)**	**Assessment of depression**	**Antidepressant class**	**Other medications**	**Comorbidities**	**MetS criteria and %**	**Main results**	**Nos**
Crichton et al. (1)	USA, 1975–2011	Cross-Sectional *N* = 970	n/a, M/F, 41%	Performed by an examiner	n/a	n/a	n/a	IDF, 44	High risk of MetS and low HDL in patients with depressive symptoms; high risk of MetS, low HDL, carbohydrate metabolism disorders (diabetes) and hypertension in patients on antidepressant therapy.	9
Hung et al. (37)	Taiwan, 2008–2009	Cross Sectional *N* = 229	44 year, M/F, 63%	n/a	SSRI—SNRI—Others	yes	n/a	IDF, 22	BMI related to MetS. Pharmacotherapy seem to be related to high BMI.	5
Kopf et al. (38)	Germany, n/a	Prospective Cohort *N* = 78	53 year, M/F, 31%	Performed by an examiner and self-administered questionnaire	SSRI—TCA	yes	no	IDF, n/a	Treatment of depression exerts a mainly beneficial effect on lipid regulation.	6
Luppino et al. (39)	Holland, 2004–2009	Cross Sectional *N* = 827	43 year, M/F, 38%	Performed by an examiner	SSRI—TCA—Others	n/a	yes	NCE-ATP III, 26	Depression severity weakly associated with glucose levels. There seem to be a mediating role for TCA and NSRI antidepressant use in increasing triglycerides levels, with limited clinical differences.	5
Margari et al. (40)	Italy, 2013–2013	Cross Sectional *N* = 160	50 y, M/F, n/a	Performed by an examiner	n/a	yes	yes	NCE-ATP III, 29	Positive association between antidepressant treatment and triglycerides and triglycerides/HDL ratio levels.	6
Sagud et al. (41)	Croatia, n/a	Cross Sectional *N* = 203	53 year, n/a, n/a	Performed by an examiner	SSRI—NRI—SNRI—Others	yes	yes	NCE-ATP III, n/a	MetS was observed in 33.5 % of patients (no significant differences between TRD and non-TRD), without correlation with cardiovascular risk factors.	5
Salvi et al. (42)	Italy, 2008–2012	Cross Sectional *N* = 289	50 year, M/F, 63%	Performed by an examiner	SSRI—TCA—SNRI—Others	yes	no	NCE-ATP III, 25	Greater frequency of MetS in patients treated with H1 high affinity antagonists.	7
Stanojević et al. (43)	Serbia, 2013–2013	Prospective Cohort *N* = 60	48 year, M/F, 52%	Performed by an examiner	SSRI	n/a	n/a	NCE-ATP III, 38	In depressed patients, elevated CRP levels associated with increased frequency of MetS.	3

*N, Number; M, Male; F, Female; y, Years; MetS, Metabolic Syndrome; n/a, not applicable; SSRI, Selective serotonin reuptake inhibitors; TCA, Tricyclic antidepressant; SNRI, Serotonin–norepinephrine reuptake inhibitors; BMI, Body Mass Index; HDL, High Density Lipoprotein; NOS, Newcastle Ottawa Scale*.

Outcome data were presented as count data. Narrative data extracted from the papers included in this mini review are reported in Table 1. Where necessary, text descriptions were used to highlight information that was not captured in the Table.

Finally, the Newcastle-Ottawa Scale (NOS) (44) was used to assess the quality of the selected cohort studies. The quality of cross sectional studies was evaluated through an adapted version of the NOS (45).

The need for an Ethics Committee approval was waived, since we just collected and synthesized data from previous clinical trials in which informed consent had already been obtained.

## Results

### Selection

**Figure 1:**
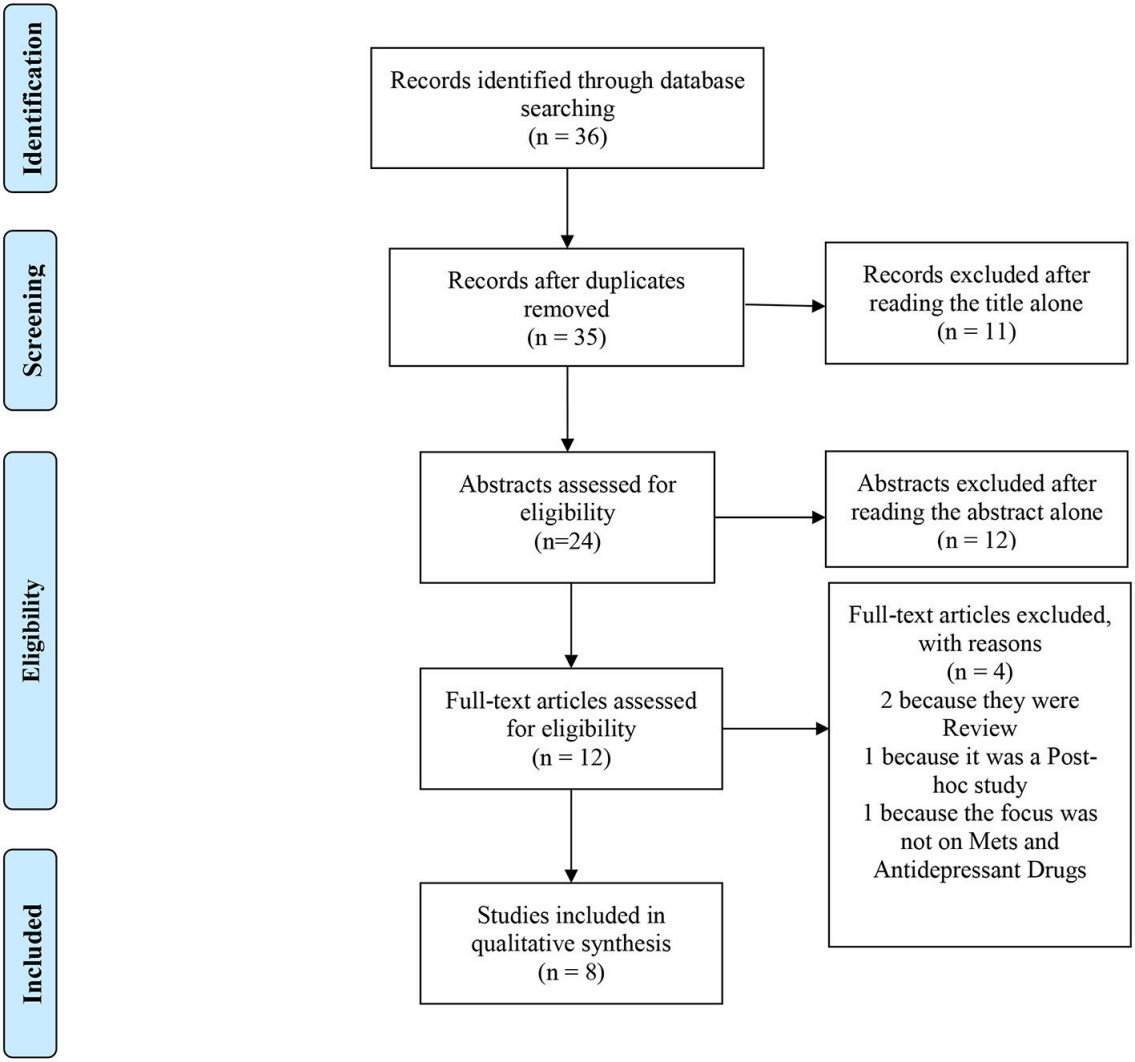
PRISMA 2009 flow diagram. Adapted from Moher et al. (47).

The PubMed literature search identified 36 articles. After title, abstract, and eventually full-text screening, 8 papers (1, 37–43) met inclusion criteria for this mini-review (see Figure 1 for more details) (46).

### Study design and features

Of the 8 studies included in our mini-review, 2 used a prospective cohort design (37, 41), and 6 a cross-sectional design (1, 38–40, 42, 43). In 3 studies the number of participants was ≤ 99 (38, 40, 43), 2 studies had a sample ranging from 100 to 240 individuals (37, 41), 3 studies included more than 250 patients (1, 39, 42). The average number of participants was of 277 ± 296 (SD) (min 60, max 970 participants). Only one study (39) was multicentric, involving 5 centers, while the other 7 ones were monocentric. Six studies were performed in Europe (38–43), 1 in the United States of America (1), and 1 in Asia (37). Only five studies reported data on the recruitment setting: specifically, two studies included inpatients from a psychiatric ward (40, 41); two studies recruited outpatients (1, 37), while the sample was mixed (both inpatients and outpatients) in 1 study (39). Three studies did not report information about recruitment setting (38, 42, 43). Only 2 studies (38, 41) reported data about the staff involved in the research, composed by doctors and nurses.

The average duration of the studies was 113 ± 180 (SD) months, ranging from a minimum of 10 to a maximum of 432 months. All the studies included in the mini review had obtained ethic committee approval.

### Socio-demographic and lifestyle features

Participants' age ranged from 18 to 98, with a mean of 47.73 ± 3.51 (SD) years. All the included studies involved participants of both genders. All studies reported sociodemographic data, which were retrieved from medical records in 6 studies (1, 38, 40–43); only one study (37) specified details about participants' occupational status. Six studies provided lifestyle data, obtained by medical records in 2 studies (38, 41), by the anamnesis in 2 other studies (40, 42), and via a specific questionnaire in the last 2 studies (1, 43)

Seven studies (1, 37, 39–43) analyzed participants' smoking habits; this information was retrieved from a direct question in 3 studies (40, 42, 43), from a self-report measure (the Nutrition and Health Questionnaire) in one study (1), and from previous-year medical records consultation in another one study (41). The source of this information was not specified in 2 studies (37, 39).

Alcohol consumption was described by all included studies. Anyway, only 1 study reported data on participants' eating habits (1), as assessed by the Nutrition and Health Information Questionnaire (32).

Two studies reported information about physical activity: 1 study (48) used the Nurses' Health Study Activity Questionnaire (49) and MET-hours per week (a metabolic equivalent is a unit that describes the energy expenditure of a specific activity) for each activity (50); 1 study reported data on structured physical activity or 30 min of walking per day (42).

### Metabolic parameters

NCE-ATP III and IDF criteria for MetS were adopted, respectively, by 5 (39–43) and 3 of the selected studies (1, 37, 38). Seven studies collected blood examinations (1, 37–43); 7 studies recorded blood pressure (1, 37, 39–43); last, 7 studies reported information about anthropometric measures (1, 37, 39–43). All the studies included in this mini-review collected data about hypertriglyceridemia (>150 mg/dl), arterial hypertension (>130/85 mmHg) and fasting hyperglycemia (>110 mg/dl); furthermore, 7 studies assessed HDL cholesterol as well (men < 40 mg/dl, women < 50 mg/dl) (37–43). Waist circumference was measured in 6 studies; in 4 of them, abdominal obesity was diagnosed for values above the following cutoffs: >102 cm for men and >88 cm for women (39, 41–43); 2 studies adopted different cutoff values: >94 cm (1), and >90 cm for men and >80 cm for women (37), respectively. Seven studies reported data on BMI (1, 37–43).

### Antidepressant medication and depressive symptoms

Six studies reported data on type of antidepressant agents (37–39, 41–43).

Six studies reported data on SSRI (37–43); only one on norepinephrine reuptake inhibitors (NRIs) (41); 4 studies on Serotonin and norepinephrine reuptake inhibitors (SNRI) (37, 39, 41, 42); 3 studies reported data on Tricyclic and tetracyclic antidepressants (TCA) (38, 39, 42); 3 studies reported data also on other drugs (37, 39, 41).

With more detail, Stanojević et al. (43) compared 35 SSRI medication-treated patients; Kopf et al. (38) analyzed 78 depressed patients in treatment with amitriptyline or paroxetine; Salvi et al. (42) involved 294 antidepressant-treated patients with bipolar disorder, treated with the use of antidepressants (SSRI, TCA, SNRI, and other medications); Hung et al. (37) analyzed different antidepressants, including paroxetine, trazodone, escitalopram, fluoxetine and venlafaxine; Luppino et al. (39) compared 302 primary care outpatients, 445 secondary care outpatients and 80 inpatients, with major depressive disorder (MDD), treated with TCA or Serotonin and norepinephrine reuptake inhibitors (SNRIs) and mirtazapine; Sagud et al. (41) analyzed patients data in therapy with SSRIs, SNRIs, tianeptine, mirtazapine, bupropion, reboxetine, and maprotiline.

Only 1 study reported information about the specific phase of MDD (41), while 4 studies included details about the severity of depressive symptoms (38–42). All studies adopted specific questionnaires for the assessment of depression (1, 38–43). In 7 studies the questionnaire was a clinician-administered interview (1, 37–39, 41–43), while in 1 study both a clinician-administered interview and a self-administered questionnaire were used (40). More specifically, 4 studies (37, 38, 41, 43) used the Hamilton Depression (HAM-D) Rating Scale (44), one (1) the CES-D scale (44), one (1) the Zung self-rating depression scale (44), another one (42) the Clinical Global Impressions Scale (CGI-BD) (51) and the Structured Clinical Interview (SCID) (52), and a last one (40) used the Brief Psychiatric Rating Scale (BPRS) (53), the HAM-D Rating Scale (44), the Spielberger State-Trait Anxiety Inventory (STAI) (53, 54) and the Personality Diagnostic Questionnaire (PDQ-4+) (53).

### Psychiatric and medical comorbidity

Four studies included participants who were treated with other psychiatric medications beyond antidepressants (37, 38, 40, 43). One study evaluated cognitive function (1). Five studies analyzed other psychiatric variables (37, 38, 40–42). Three studies reported information about medical comorbidities (39, 41), and 2 studies about treatment for medical conditions beyond depression (40, 41).

### Outcomes and assessment of the quality of studies

Stroke was reported in 1 study (1), Cardiovascular Disease (CVD) in 3 studies (37, 39, 41). The NOS scores ranged from 3 to 9, with a mean score of 5.75 ± 1.75 (SD).

### Cross-sectional studies

Sagud et al. (41) assessed 203 inpatients with MDD, including both treatment resistant (TRD) and non-treatment resistant (non-TRD) individuals. They did not find any relationship between MetS and treatment resistance.

Crichton et al. (1) suggested that cardiovascular risk factors attenuated the relation between depression and MetS; moreover they shown that, while depression appeared to increase the risk of CVD (1), CVD could promote the onset of depression.

The cross-sectional study by Salvi et al. (42) involved 294 antidepressant-treated patients with bipolar disorder. No association was found between the use of antidepressants (SSRI, TCA, SNRI, and others) and MetS. However, subjects on H1-R high-affinity antidepressants treatment (*N* = 15) showed a higher prevalence of MetS, likely due to the anti-histaminic effect of this type of antidepressants, which counteracts histamine central anorexigenic effects (6) and increases adipose tissue deposition (8).

Luppino et al. (39) compared 302 primary care outpatients, 445 secondary care outpatients and 80 inpatients with MDD. No significant difference among patients recruited in the three treatment settings was found either in the prevalence of MetS (26% primary, 24% secondary care, and 28% inpatients) or in clinical and laboratory measures including waist circumference (WC), BMI, LDL cholesterol, glucose and diastolic blood pressure (DBP). However, inpatients reported higher waist-hip ratio, total cholesterol and triglyceride levels and lower HDL cholesterol levels and systolic blood pressure than outpatients. Results showed significant associations for TCA use with higher DBP (β = 0.10, *P* = 0.003) and LDL-cholesterol (β = 0.07, *P* = 0.04), while the use of other antidepressants (Serotonin and norepinephrine reuptake inhibitors-SNRIs and mirtazapine) was associated with higher triglyceride levels (β = 0.10, *P* = 0.004).

Hung et al. (37) studied 229 outpatients, 85 males and 144 females, recruited by systematic sampling of 1,147 outpatients affected by anxiety and mood disorders. The authors analyzed the impact of pharmacotherapy and psychiatric diagnoses on MetS and found that 51 (22.3%) subjects of 229 outpatients matched MetS criteria, likely due to treatment with antipsychotics and mood stabilizers. The study also shown that antidepressant-treated (paroxetine, trazodone, duloxetine, escitalopram, fluoxetine, and venlafaxine) patients and patients treated with other medication than-antidepressants (antipsychotics and mood stabilizers) did not significantly differ as far as MetS risk is concerned.

Margari et al. (40) evaluated the differences in anthropometric measures, biochemical variables, MetS and cardiovascular risk in a sample of 83 psychiatric inpatients under pharmacological treatment and 77 internal medicine patients. Female psychiatric patients showed higher levels of triglycerides (mg) and of homeostatic model assessment (HOMA) index than males. Patients with unipolar depression reported highest triglycerides and triglycerides/HDL ratio levels with a strong relation with antidepressant treatment.

### Cohort studies

Stanojević et al. (43) compared 35 SSRI medication-treated patients with recurrent depressive disorder and 25 healthy controls. Elevated C-reactive protein (CRP) levels were found to be associated with an increased frequency of MetS in depressed patients. While no statistically significant difference was found between depressed patients and controls regarding either the prevalence of MetS or CRP levels, waist circumference and total cholesterol levels were significantly higher in the first than in the latter.

Kopf et al. (38) analyzed lipoprotein composition, insulin sensitivity and salivary cortisol in 78 depressed patients in treatment with amitriptyline or paroxetine at baseline (t0) and after 35 days of treatment. No change in quantitative insulin sensitivity check index (QUICKI) was found after 35 days of treatment. Moreover, only patients on amitriptyline treatment showed increased levels of triglycerides (*P* < 0.05). Parameters of cholesterol metabolism improved slightly without differences between treatment groups. The authors concluded that both the antidepressant treatments assessed might exert a mainly beneficial effect on lipid regulation.

### Narrative analysis

A summary of extracted data is reported in Table 1.

## Discussion

Previous studies suggested a higher risk of unhealthy behaviors (i.e., excessive alcohol consumption, smoking, obesity, lack of physical activity) (55, 56) in depressed patients, both with a current depressive episode or with a lifetime diagnosis of depression (57), which may contribute to their increased risk of MetS. Moreover, severity of depression seems to correlate with smoking, obesity and physical inactivity following a dose–response mechanism (58–60). Actually, Pan and coworkers' systematic review (18) described an association between depression and MetS in adults and supported early detection and management of depression among patients with MetS and vice versa.

The current mini review included two cohort (37, 41) and six cross-sectional studies (1, 38–40, 42, 43). Most of them were performed in Europe and involved a single center. Patients were recruited from different settings, including psychiatric wards, outpatient services or both. Six studies reported data on the type of antidepressant assessed (37–39, 41–43), and 6 studies included patients treated with other psychiatric medications beyond antidepressants (37, 38, 40–43).

As regards correlation between MetS and antidepressants use, only one study (42) showed that the risk of Mets was greater for patients treated with antidepressants with high affinity binding to histamine H1-receptors. There seems to be an association between the use of antidepressant medication and alterations of lipid profile. In particular, Luppino et al. (39) suggested a mediating role of TCA and NSRI antidepressant use on the increase of triglycerides level (40), while Crichton et al. (1) suggested a correlation with carbohydrate metabolism disorders (diabetes) and hypertension in patients on antidepressant therapy. Hung et al. (37) postulated that pharmacotherapy may lead to over-weight problems.

The studies included in this mini review, consistent with a previous report on this topic (61), support the hypothesis that, beyond the use of antidepressant medications, MetS may be associated with depressive symptoms, especially when associated with neurovegetative features, and that the inflammatory response may play a key role in this phenomenon. Indeed, the study by Stanojević et al. (43) found that elevated CRP levels in depressed patients were associated with an increased frequency of MetS.

Two studies included in the mini review (1, 39) support the idea that the increased rate of Mets in depressed patients might depend on the involvement of both depressive symptoms and antidepressants use. In particular, one study (1) showed that, among patients with depressive symptoms and lower HDL rates, there seems to be a higher risk of MetS Sagud et al. (41) did not find any significant difference between TRD and non-TRD in the occurrence of MetS or cardiovascular risk factors.

Only the cross sectional study by Kopf et al. (38), observed an inverse correlation between cortisol and cholesterol in the obese subgroup of amitriptyline or paroxetine-treated patients, consistent with the widely acknowledged association of depression and low cholesterol levels (9, 10), and supporting the hypothesis that antidepressants may exert a mainly beneficial effect on lipid regulation.

Moreover, studies using IDF criteria reported stronger correlations among depression and Mets than those using NCEP ATP-III ones (40–42) with the exception of the study by Kopf et al. (38).

### Strengths

Our mini review followed the Preferred Reporting Items for Systematic Reviews and Meta-Analyses (PRISMA) statement (46). Quality of included studies was assessed with the NOS (NOS scores ranged from 3 to 9). Accuracy of MEDLINE search was guaranteed by using MeSH terms and text words related to research studies on association between MetS, depression and antidepressants.

## Limits

The mini review was limited to publications in English language available in the MEDLINE database. The choice of a simple single search query might have caused the inclusion of a limited number of articles in the mini review. Therefore, it is possible that the literature search we performed was not comprehensive, and that excluding other electronic databases and languages other than English may have caused the exclusion of potentially interesting articles. Nonetheless, recently it has been suggested that there is no significant effect on the outcome of a review including only one database rather than more than one (62, 63).

## Conclusions

The results of this mini review seem to slightly support the association among depressive symptoms, antidepressants therapy and MetS. Nonetheless, due to the absence of reliable and detailed trial data reported in the studies included in the review, it could be difficult to tease out effects of depression from those of the medications used.

Overall, antidepressants do not seem clearly associated with MetS, except for H1-R high-affinity ones. Notwithstanding the limitations described above and the heterogeneity of the selected studies (i.e., study design, sample size, analysis, participants' and setting features, classification criteria of depression and MetS, different antidepressant drugs), implications seem to emerge especially for antidepressant-treated patients with depressive symptoms, receiving also antipsychotics or mood stabilizers, who should be carefully monitored for MetS and the correlated potentially life-threatening clinical conditions (such as diabetes/CVD).

Prevention strategies, early diagnosis, integrated and collaborative health care systems should be available for patients with MetS and depression; treatment and lifestyle changes should be considered in high-risk patients.

## Author contributions

CG and PZ contributed to the conception and design of the work, with decision about search string used on Pubmed database. EG and CG developed and implemented the methods of the manuscript. EG and CG independently triaged the titles and abstracts identified by the search to remove those that were clearly inappropriate. The remaining papers, to be included, had to satisfy all the predetermined eligibility criteria. Possible disagreements regarding study inclusion were resolved by discussion with PZ. After selection of the relevant studies, PC and MC-S independently extracted and tabulated data on study design and outcome data using a standard form. GB, LL, and EG prepared the manuscript. EG performed statistical analysis. CG, EG, and PZ revised it critically for important intellectual content.

### Conflict of interest statement

The authors declare that the research was conducted in the absence of any commercial or financial relationships that could be construed as a potential conflict of interest.

## References

[1] Crichton GE Elias MF Robbins MA. Association between depressive symptoms, use of antidepressant medication and the metabolic syndrome: the maine-syracuse study. BMC Public Health. (2016) 16:1–9. 10.1186/s12889-016-3170-2 27287001 PMC4902917

[2] Chokka P Tancer M Yeragani VK. Metabolic syndrome: relevance to antidepressant treatment. J Psychiatry Neurosci. (2006) 31:414. 17136222 PMC1635794

[3] Alberti KGMM Eckel RH Grundy SM Zimmet PZ Cleeman JI Donato KA. Harmonizing the metabolic syndrome: a joint interim statement of the international diabetes federation task force on epidemiology and prevention; National heart, lung, and blood institute; American heart association; World heart federation; International. Circulation. (2009) 120:1640–5. 10.1161/CIRCULATIONAHA.109.192644 19805654

[4] IDF. The IDF consensus worldwide definition of the metabolic syndrome. Available online at: http://www.idf.org/webdata/docs/IDF_Meta_def_final.pdf (Accessed June 11, 2011).

[5] Kubrusly M de Oliveira CMC Simões PSF Lima R de O Galdino PNR Sousa P de AF. Prevalence of metabolic syndrome according to NCEP-ATP III and IDF criteria in patients on hemodialysis. J Bras Nefrol. (2015) 37:72–8. 10.5935/0101-2800.20150011 25923753

[6] Jorgensen EA Knigge U Warberg J Kjaer A. Histamine and the regulation of body weight. Neuroendocrinology (2007) 86:210–4. 10.1159/000108341 17848791

[7] Vogt BP Souza PL Minicucci MF Martin LC Barretti P Caramori JT. Metabolic syndrome criteria as predictors of insulin resistance, inflammation and mortality in chronic hemodialysis patients. Metab Syndr Relat Disord. (2014) 12:443–9 10.1089/met.2014.0011 25099153

[8] He M Zhang Q Deng C Wang H Lian J Huang XF. Hypothalamic histamine H1 receptor-AMPK signaling time-dependently mediates olanzapine-induced hyperphagia and weight gain in female rats. Psychoneuroendocrinology (2014) 42:153–64. 10.1016/j.psyneuen.2014.01.018 24636512

[9] Opie RS O'Neil A Itsiopoulos C Jacka FN. The impact of whole-of-diet interventions on depression and anxiety: a systematic review of randomised controlled trials. Public Health Nutr. (2015) 18:2074–93. 10.1017/S1368980014002614 25465596 PMC10271872

[10] Vilibić M Jukić V PandŽić-Sakoman M Bilić P Milošević M. Association between total serum cholesterol and depression, aggression, and suicidal ideations in war veterans with posttraumatic stress disorder: a cross-sectional study. Croat Med J. (2014) 55:520–9. 10.3325/cmj.2014.55.520 25358885 PMC4228297

[11] Grundy SM Brewer HB Cleeman JI Smith SC Lenfant C. Definition of metabolic syndrome: report of the national heart, lung, and blood institute/American heart association conference on scientific issues related to definition. Circulation (2004) 109:433–8. 10.1161/01.CIR.0000111245.75752.C6 14744958

[12] Roshanaei-Moghaddam B Katon W. Premature mortality from general medical illnesses among persons with bipolar disorder: a review. Psychiatr Serv. (2009) 60:147–56. 10.1176/ps.2009.60.2.147 19176408

[13] Newcomer JW. Metabolic syndrome and mental illness. Am J Manag Care (2007) 13(Suppl. 7):S170–7. 18041878

[14] Narasimhan M RJ Evidence-based perspective on metabolic syndrome and use of antipsychotics. Drug Benefit Trends. (2010) 22:77–88.

[15] DE Hert M Correll CU Bobes J Cetkovich-Bakmas M Cohen D Asai I. Physical illness in patients with severe mental disorders. I. Prevalence, impact of medications and disparities in health care. World Psychiatry. (2011) 10:52–77. 10.1002/j.2051-5545.2011.tb00014.x 21379357 PMC3048500

[16] Joukamaa M Heliovaara M Knekt P Aromaa A Raitasalo R Lehtinen V. Mental disorders and cause-specific mortality. Br J Psychiatry (2001) 179:498–502. 10.1192/bjp.179.6.498 11731351

[17] Lopuszanska U Skorzynska-Dziduszko K Lupa-Zatwarnicka K Makara-Studzinska M. Mental illness and metabolic syndrome – a literature review. Ann Agric Environ Med. (2014) 21:815–21. 10.5604/12321966.1129939 25528926

[18] Rojo LE Gaspar PA Silva H Risco L Arena P Cubillos-Robles K. Metabolic syndrome and obesity among users of second generation antipsychotics: a global challenge for modern psychopharmacology. Pharmacol Res. (2015) 101:74–85. 10.1016/j.phrs.2015.07.022 26218604

[19] Klingerman CM Stipanovic ME Hajnal A Lynch CJ. Acute metabolic effects of olanzapine depend on dose and injection site. Dose Response (2015) 13:1–8. 10.1177/1559325815618915 26740814 PMC4679189

[20] Pan A Keum N Okereke OI Sun Q Kivimaki M Rubin RR. Bidirectional association between depression and metabolic syndrome: a systematic review and meta-analysis of epidemiological studies. Diabetes Care (2012) 35:1171–80. 10.2337/dc11-2055 22517938 PMC3329841

[21] Vancampfort D Correll CU Wampers M Sienaert P Mitchell AJ De Herdt A. Metabolic syndrome and metabolic abnormalities in patients with major depressive disorder: a meta-analysis of prevalences and moderating variables. Psychol Med. (2014) 44:2017–28. 10.1017/S0033291713002778 24262678

[22] Rhee SJ Kim EY Kim SH Lee HJ Kim B Ha K. Subjective depressive symptoms and metabolic syndrome among the general population. Prog Neuropsychopharmacol Biol Psychiatry (2014) 54:223–30. 10.1016/j.pnpbp.2014.06.006 24975752

[23] Rethorst CD Bernstein I Trivedi MH. Inflammation, obesity and metabolic syndrome in depression: analysis of the 2009–2010 National Health and Nutrition Survey (NHANES). J Clin Psychiatry. (2015) 75:1–14. 10.4088/JCP.14m09009 25551239 PMC4309548

[24] Salvi V Grua I Cerveri G Mencacci C Barone-Adesi F. The risk of new-onset diabetes in antidepressant users – a systematic review and meta-analysis. PLoS ONE (2017) 12:1–14. 10.1371/journal.pone.0182088 28759599 PMC5536271

[25] Eckel RH Alberti KG Grundy SM Zimmet PZ. The metabolic syndrome. Lancet (2010) 375:181–3. 10.1016/S0140-6736(09)61794-3 20109902

[26] Foley DL Morley KI Madden PA Heath AC Whitfield JB Martin NG. Major depression and the metabolic syndrome. Twin Res Hum Genet. (2010) 13:347–8. 10.1375/twin.13.4.347 20707705 PMC3150840

[27] Hiles SA Révész D Lamers F Giltay E Penninx BWJH. Bidirectional prospective associations of metabolic syndrome components with depression, anxiety, and antidepressant use. Depress Anxiety (2016) 33:754–64. 10.1002/da.22512 27120696 PMC5111740

[28] Janssen DGA Caniato RN Verster JC Baune BT. A psychoneuroimmunological review on cytokines involved in antidepressant treatment response. Hum Psychopharmacol. (2010) 25:201–15. 10.1002/hup.1103 20373471

[29] Bersani FS Lindqvist D Mellon SH Penninx BWJH Verhoeven JE Revesz D. Telomerase activation as a possible mechanism of action for psychopharmacological interventions. Drug Discov Today (2015) 20:1305–9. 10.1016/j.drudis.2015.06.016 26166813

[30] Mojtabai R Olfson M. National trends in long-term use of antidepressant medications: results from the U.S. National Health and Nutrition Examination Survey. J Clin Psychiatry (2014) 75:169–77. 10.4088/JCP.13m08443 24345349

[31] Blumenthal SR Castro VM Clements CC Rosenfield HR Murphy SN Fava M. An electronic health records study of long-term weight gain following antidepressant use. JAMA Psychiatry (2014) 71:889–96. 10.1001/jamapsychiatry.2014.414 24898363 PMC9980723

[32] Johnson CF Williams B Macgillivray SA Dougall NJ Maxwell M “Doing the right thing”: factors influencing GP prescribing of antidepressants and prescribed doses. BMC Fam Pract. (2017) 18:1–13. 10.1186/s12875-017-0643-z 28623894 PMC5473964

[33] Beyazyuz M Albayrak Y Egilmez OB Albayrak N Beyazyuz E. Relationship between SSRIs and metabolic syndrome abnormalities in patients with generalized anxiety disorder: a prospective study. Psychiatry Investig. (2013) 10:148–54. 10.4306/pi.2013.10.2.148 23798963 PMC3687049

[34] van Reedt Dortland AKB Giltay EJ van Veen T Zitman FG Penninx BWJH. Metabolic syndrome abnormalities are associated with severity of anxiety and depression and with tricyclic antidepressant use. Acta Psychiatr Scand. (2010) 122:30–9. 10.1111/j.1600-0447.2010.01565.x 20456284

[35] McIntyre RS Park KY Law CWY Sultan F Adams A Lourenco MT. The association between conventional antidepressants and the metabolic syndrome: a review of the evidence and clinical implications. CNS Drugs (2010) 24:741–53. 10.2165/11533280-000000000-00000 20806987

[36] Raeder MB Bjelland I Emil Vollset S Steen VM. Obesity, dyslipidemia, and diabetes with selective serotonin reuptake inhibitors: the hordaland health study. J Clin Psychiatry (2006) 67:1974–82. 10.4088/JCP.v67n1219 17194277

[37] Hung C-I Liu C-Y Hsiao M-C Yu N-W Chu C-L. Metabolic syndrome among psychiatric outpatients with mood and anxiety disorders. BMC Psychiatry (2014) 14:185. 10.1186/1471-244X-14-185 24952586 PMC4079179

[38] Kopf D Westphal S Luley CW Ritter S Gilles M Weber-Hamann B. Lipid metabolism and insulin resistance in depressed patients: Significance of weight, hypercortisolism, and antidepressant treatment. J Clin Psychopharmacol. (2004) 24:527–31. 10.1097/01.jcp.0000138762.23482.63 15349009

[39] Luppino FS Bouvy PF Giltay EJ Penninx BWJH Zitman FG. The metabolic syndrome and related characteristics in major depression: inpatients and outpatients compared metabolic differences across treatment settings. Gen Hosp Psychiatry (2014) 36:509–15. 10.1016/j.genhosppsych.2014.05.018 25001528

[40] Margari F Zagaria G Lozupone M Minerva F Pisani R Palasciano G. Metabolic syndrome: differences between psychiatric and internal medicine patients. Int J Psychiatry Med. (2013) 45:203–26. 10.2190/PM.45.3.a 24066405

[41] Sagud M Mihaljevic-Peles A Uzun S Cusa BV Kozumplik O Kudlek-Mikulic S. The lack of association between components of metabolic syndrome and treatment resistance in depression. Psychopharmacology (2013) 230:15–21. 10.1007/s00213-013-3085-x 23579429

[42] Salvi V Barone-Adesi F D'Ambrosio V Albert U Maina G. High H1-affinity antidepressants and risk of metabolic syndrome in bipolar disorder. Psychopharmacology (2016) 233:49–56. 10.1007/s00213-015-4085-9 26407602

[43] Stanojević A Popović I Nenadović M Ravanić D Paunović-Milosavljević G. Metabolic syndrome and C-reactive protein in patients with depressive disorder on antidepressive medication. Srp Arh Celok Lek. (2013) 141:511–5. 24073559 10.2298/sarh1308511s

[44] Wells G Shea B O'Connell D Peterson J Welch V Losos M Newcastle-ottawa quality assessment form for cohort studies. Ottawa Hosp Res Inst. (2014) 17–8.

[45] Modesti PA Reboldi G Cappuccio FP Agyemang C Remuzzi G Rapi S. Panethnic differences in blood pressure in Europe: a systematic review and meta-analysis. PLoS ONE (2016) 11:e0147601. 10.1371/journal.pone.0147601 26808317 PMC4725677

[46] Liberati A Altman DG Tetzlaff J Mulrow C Gøtzsche PC Ioannidis JPA. The PRISMA statement for reporting systematic reviews and meta-analyses of studies that evaluate health care interventions: explanation and elaboration. PLoS Med. (2009):6:b2700. 10.1136/bmj.b2700 19621070 PMC2707010

[47] Moher D Liberati A Tetzlaff J Altman DG Altman D Antes G Preferred reporting items for systematic reviews and meta-analyses: the PRISMA statement. PLoS Med. (2009) 6:e1000097 10.1371/journal.pmed.1000097 19621072 PMC2707599

[48] Crichton GE Elias MF Davey A Alkerwi A. Cardiovascular health and cognitive function: the maine-syracuse longitudinal study. PLoS ONE (2014) 9:1–9. 10.1371/journal.pone.0089317 24595096 PMC3940600

[49] Wolf AM Hunter DJ Colditz GA Manson JE Stampfer MJ Corsano KA. Reproducibility and validity of a self-administered physical activity questionnaire. Int J Epidemiol. (1994) 23:991–9. 10.1093/ije/23.5.991 7860180

[50] Ainsworth BE Haskell WL Leon AS Jacobs DR Montoye HJ Sallis JF. Compendium of physical activities: classification of energy costs of human physical activities. Med Sci Sport Exerc. (1993) 25:71–80 10.1249/00005768-199301000-00011 8292105

[51] Busner J Targum SD. Global Impressions Scale: applying a research. Psychiatry (2007) 4:29–37. 20526405 PMC2880930

[52] Drill R Nakash O DeFife JA Westen D. Assessment of clinical information: comparison of the validity of a Structured Clinical Interview (the SCID) and the Clinical Diagnostic Interview. J Nerv Ment Dis. (2015) 203:459–62. 10.1097/NMD.0000000000000300 25974055 PMC4452387

[53] Beller SA Overall JE. The Brief Psychiatric Rating Scale (BPRS) in geropsychiatric research: II. Representative profile patterns. J Gerontol. (1984) 39:194–200. 10.1093/geronj/39.2.194 6699375

[54] Kvaal K Ulstein I Nordhus IH Engedal K. The spielberger state-trait anxiety inventory (STAI): the state scale in detecting mental disorders in geriatric patients. Int J Geriatr Psychiatry (2005) 20:629–34. 10.1002/gps.1330 16021666

[55] Allgower A Wardle J Steptoe A. Depressive symptoms, social support, and personal health behaviors in young men and women. Health Psychol. (2001) 20:223–7. 10.1037/0278-6133.20.3.223 11403220

[56] Fergusson DM Goodwin RD Horwood LJ. Major depression and cigarette smoking: results of a 21-year longitudinal study. Psychol Med. (2003) 33:1357–67. 10.1017/S0033291703008596 14672244

[57] Martinsen EW. Physical activity and depression: clinical experience. Acta Psychiatr Scand Suppl. (1994) 377:23–7. 10.1111/j.1600-0447.1994.tb05797.x 8053362

[58] Strine TW Mokdad AH Dube SR Balluz LS Gonzalez O Berry JT. The association of depression and anxiety with obesity and unhealthy behaviors among community-dwelling US adults. Gen Hosp Psychiatry (2008) 30:127–37. 10.1016/j.genhosppsych.2007.12.008 18291294

[59] Ludwig VM Bayley A Cook DG Stahl D Treasure JL Asthworth M. Association between depressive symptoms and objectively measured daily step count in individuals at high risk of cardiovascular disease in South London, UK: a cross-sectional study. BMJ Open (2018) 8:e020942. 10.1136/bmjopen-2017-020942 29654044 PMC5898324

[60] Okoro CA Stoodt G Rohrer JE Strine TW Li C Balluz LS. Physical activity patterns among U.S. adults with and without serious psychological distress. Public Health Rep. (2014) 129:30–8. 10.1177/003335491412900106 24381357 PMC3863001

[61] Capuron L Su S Miller AH Bremner JD Goldberg J Vogt GJ. Depressive symptoms and metabolic syndrome: is inflammation the underlying link? Biol Psychiatry (2008) 64:896–900. 10.1016/j.biopsych.2008.05.019 18597739 PMC2621309

[62] Rice DB Kloda LA Levis B Qi B Kingsland E Thombs BD. Are MEDLINE searches sufficient for systematic reviews and meta-analyses of the diagnostic accuracy of depression screening tools? a review of meta-analyses. J Psychosom Res. (2016) 87:7–13. 10.1016/j.jpsychores.2016.06.002 27411746

[63] van Enst WA Scholten RJ Whiting P Zwinderman AH Hooft L. Meta-epidemiologic analysis indicates that MEDLINE searches are sufficient for diagnostic test accuracy systematic reviews. J Clin Epidemiol. (2014) 67:1192–9. 10.1016/j.jclinepi.2014.05.008 24996667

